# Bibliography

**Published:** 1916-09

**Authors:** 


### BIBLIOGRAPHY

PROFITABLE PRACTICE. This is a book devoted exclusively to the business side of dentistry and is written by Dr. George Wood Clapp, editor of the *Dental Digest.* Much of the material has appeared in that journal over the nom de plum of "Brother Bill." Dr. Clapp has the view of dentistry as a profession that it is also a business. His motto seems to be that "Dentistry as a means of service is a profession, as a means of livelihood it is a business." To use a slang expression it is a kind of "fifty-fifty" affair. The book is filled with business suggestions from cover to cover and one can hardly criticise them as such, only it makes one feel that the high ideals which every true professional man sooner or later erects for himself are drowned out in a flood of commercialism. The book shows a vast amount of thoughtful consideration of the business side of a dental practice and no one could read it without getting ideas that would be invaluable in establishing correct methods of business dealings.

Some ideas in the book will not appeal strongly to the ethically inclined. If a patient needs a certain service that is more expensive than he can pay for, a dentist is justified from the good business viewpoint, in making the operation partially or in a less expensive manner. Of course that would be sensible so long as the less expensive method would not work an injury to the patient, but who can say that at some time an abbreviated service may not become a vicious one, surely no one would be justified in taking chances where results may be of vital importance because a compensatory fee was not forthcoming. The author cites the case of a physician who refused to complete an X-ray diagnosis unless the patient could pay $150. If the X-ray diagnosis was really necessary that physician did not measure up to his privilege to say nothing of his duty. If the "Good Samaritan" had done as the "Priest" and

"Levite" did the case would have been parallel. The true Samaritan physician not only carried the injured man to the hotel but paid his bill before he went on. If we are to nail every professional service down to a hard and fast price how can we call ourselves servants, and what becomes of our boasted professionalism? There are many statements like this in this book that seem inconsistent with true professional ideals, and we fear the consequences of such an attitude for its influence on young men just entering the profession and also for the justification it may be to such as are exploiting the profession already too much on the business basis.

The book has a lot of useful information in it and can be used to great advantage by any one who is careless or indifferent to correct business methods. We believe, however, that it could have been tempered with professional ideals of the traditional type and be a much stronger and more useful contribution to this most important function of a professional man's career. It is published by the Dentists' Supply Co., of New York.

